# Haptic Feedback Systems for Lower-Limb Prosthetic Applications: A Review of System Design, User Experience, and Clinical Insights

**DOI:** 10.3390/bioengineering12090989

**Published:** 2025-09-18

**Authors:** Mohammadmahdi Karimi, Nashmin Yeganeh, Ivan Makarov, Atli Örn Sverrisson, Karl Fannar Gunnarsson, Kristín Briem, Sigurður Brynjólfsson, Árni Kristjánsson, Runar Unnthorsson

**Affiliations:** 1 School of Engineering and Natural Sciences, University of Iceland, 102 Reykjavík, Iceland; nay2@hi.is (N.Y.); sb@hi.is (S.B.); runson@hi.is (R.U.); 2 School of Health Sciences, University of Iceland, 102 Reykjavík, Iceland; ivm3@hi.is (I.M.); karlfg@hi.is (K.F.G.); kbriem@hi.is (K.B.); ak@hi.is (Á.K.); 3 Össur Ehf., Grjothals 1–5, 110 Reykjavík, Iceland; 4 Landspítali—National University Hospital, 105 Reykjavík, Iceland

**Keywords:** electrotactile, gait symmetry, mechanotactile, proprioception, prostheses, prosthetic rehabilitation, sensory feedback, spatial acuity, vibrotactile, wearable technology

## Abstract

Systems presenting haptic information have emerged as an important technological advance in assisting individuals with sensory impairments or amputations, where the aim is to enhance sensory perception or provide sensory substitution through tactile feedback. These systems provide information on limb positioning, environmental interactions, and gait events, significantly improving mobility in amputees and their confidence about using such devices. This review summarizes recent progress in haptic feedback systems by providing a comparative analysis of different feedback approaches, evaluating their clinical effectiveness and usability, tactile feedback system design, and user experience, while identifying key gaps in the literature. These insights can contribute to the advancement of more effective, user-centered haptic feedback systems tailored for lower limb prosthetics. The findings are aimed at guiding future research in designing adaptive, intuitive, and clinically viable feedback mechanisms, fostering the widespread implementation of haptic systems in both assistive and rehabilitative applications.

## 1. Introduction

Amputation of the lower-limb and the associated loss of sensory and motor function adversely affects proprioception [1], balance [2], and gait [3], greatly complicating daily activities. The resulting compensatory movement patterns increase the physical load on the remaining limbs, with negative long-term effects on an amputee’s mobility [4,5,6] and health-related quality of life [7,8,9]. Various haptic feedback mechanisms have been developed that provide real-time information through vibrotactile, electrotactile, and mechanotactile stimuli, each offering unique advantages and challenges (Table 1). Due to the rapid advancement of prosthetic technologies [10,11,12,13,14], tactile feedback systems have been introduced as a viable method to counter sensory loss and provide amputees with some aspects of normal sensory feedback [15,16].

**Figure 1:**
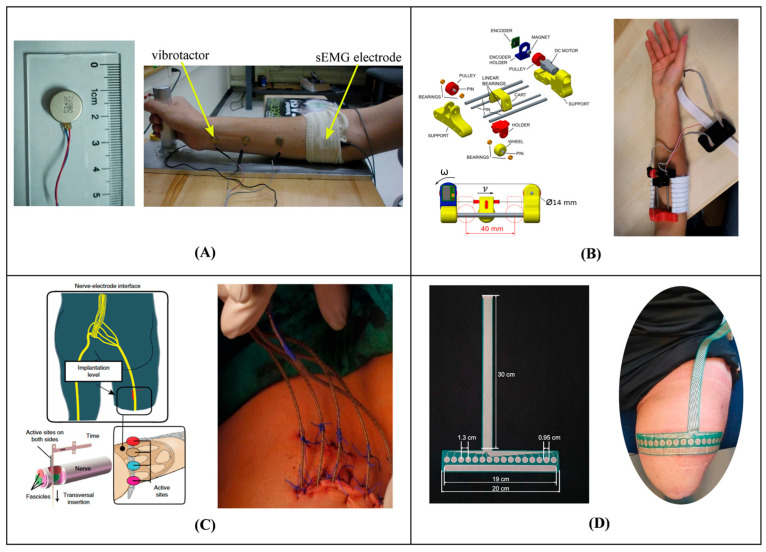
Sensory substitution: (**A**) Vibrator (**left**) [26]; vibrotactile stimulation on the forearm with surface electromyography (sEMG) electrodes (**right**) [27]. (**B**) Mechanotactile stimulation on the forearm [20]. (**C**) Intraneural sensory feedback on a residual limb [28]. (**D**) Electrotactile stimulation on a residual limb [29].

The basic concept behind sensory substitution is to replace or improve deficient sensory inputs through alternative pathways [17]. Within the area of prosthetics, haptic feedback systems deliver real-time sensory feedback via vibrational, electrical or mechanical stimuli to transmit information regarding the prosthesis. For lower limb amputees, this feedback can markedly enhance joint proprioception and improve postural control [18], allowing users to experience the movement and alignment of their prosthetic limb in space. For individuals with amputations, vibrotactile feedback applied to the skin has proven effective to provide information about artificial limbs. This form of feedback allows users to perceive contact with the environment and better control their movements, ultimately bridging the sensory gap created by limb loss [18,19]. Other haptic methods add further versatility and precision to the sensory experience, catering to diverse user needs and preferences (Figure 1). These methods include mechanotactile stimulation [20], which applies physical forces such as pressure, skin stretch, or localized mechanical displacement to the skin through actuators, and electrotactile feedback [21], which delivers controlled electrical impulses to the skin via electrodes to stimulate sensory nerves and create artificial touch perceptions by modulating current intensity, frequency, and pulse width.

Lower-limb amputees often express a lack of confidence in their prosthetic devices. Navigating uneven terrain, negotiating stairs and slopes, and maintaining balance in crowded or dimly lit environments remain significant challenges and contribute to a heightened risk of falls, which can further restrict activity and social participation [22]. Neuroprosthetic systems, which aim to integrate intent control and sensory feedback, are being developed to bridge this functionality gap. By providing real-time feedback and improving the responsiveness of prosthetic limbs, these systems have the potential to not only enhance physical mobility but also reduce cognitive load and increase trust in the prosthetic device [23]. Research on sensory substitution indicates that tactile feedback can lower the risk of falls [24], enhance gait efficiency, and improve walking symmetry [25], significantly improving benefits from lower-limb prosthetics.

Furthermore, users’ interactions with their prosthesis are significantly impacted by haptic feedback, as sensory stimuli augment the sense of embodiment and psychological reassurance when utilizing their prosthesis [30]. Some users feel that haptic feedback systems cause the prosthesis to feel like a natural body extension. This phenomenon is similar to what has been termed externalization [17] or distal attribution [31] in the sensory substitution literature, where sensory input from the prosthesis is experienced not as skin-level stimulation but as originating from the external environment. This perceptual shift, often enabled through training and sensorimotor coupling, supports the integration of prosthetic feedback into the user’s body schema, thereby alleviating the cognitive load associated with device operation and facilitating more spontaneous and intuitive interactions with their environment [32]. By fostering a sense of connection and ownership over the prosthetic limb, haptic feedback contributes not only to functional performance but also to improved mental well-being and confidence [33].

While some reviews have recently explored sensory or biofeedback in prosthetics, their scope and emphasis have distinct differences from the proposed review. Esca-milla-Nunez et al. [34] concentrated on gait rehabilitation across modalities without delving into the engineering of haptic systems, while Raspopovic et al. [35] had a strong focus on invasive neural interfaces, and Masteller et al. [36] addressed only upper-limb feedback systems. In contrast, this narrative review will present the latest research on the design, implementation, and benefits of haptic feedback systems, particularly in their applications for individuals with lower limb amputations. Contributions from various authors will be explored on the topics of diverse feedback systems, optimal tactor spacing and frequency, spatial acuity, user comfort, and learning effects in wearable technology. Despite advances in haptic feedback systems, challenges remain in optimizing feedback delivery, ensuring user adaptability, and validating long-term usability. The aim is therefore to highlight the most recent advances in sensory feedback systems, offering a comprehensive understanding of how haptic feedback systems can be most efficiently used for sensory substitution and prosthetic rehabilitation.

## 2. Materials and Methods

This review was conducted using a narrative approach to identify knowledge gaps, key advances, practical applications, and challenges in haptic feedback systems for prosthetics. Sources were selected from a combination of academic databases, including Scopus, ScienceDirect (Elsevier, Amsterdam, The Netherlands), Web of Science, PubMed and IEEE Xplore. General search terms such as “haptic feedback systems,” “vibrotactile,” “mechanotactile,” “electrotactile,” “prosthetic sensory feedback,” and “sensory substitution” guided the exploration. The selection process prioritized relevance, recency, and the availability of detailed findings related to haptic feedback for prosthetics. A snowballing search strategy was used to identify additional relevant studies by examining the references and citing lists of key papers.

The search was broad and exploratory rather than exhaustive, aiming to capture a diverse range of perspectives and technological approaches, focusing on three central themes that are presented in sections below, relating to clinical and functional outcomes, system design principles, and user experience. Key findings were charted and synthesized to emphasize significant trends, recurring challenges, and innovative solutions. Although the review does not follow a systematic methodology, efforts were made to include diverse and representative sources to provide a comprehensive understanding of haptic feedback systems in prosthetic applications. Because of the narrative nature of the review, no strict inclusion or exclusion criteria were applied. Consequently, database-specific breakdowns regarding, e.g., article counts or Boolean operator strategies are not provided.

## 3. Clinical and Functional Outcomes

The literature shows that sensory substitution systems using haptic feedback most often use vibrotactile or electrotactile feedback (Figure 1) with the aim of reducing cognitive load and improving overall prosthesis control.

### 3.1. Gait Symmetry

Asymmetry is commonly seen in lower-limb amputee gait [3,37,38], and compensatory strategies may put strain on the intact limb [39]. Studies by Dietrich et al. [40] and Crea et al. [41] have demonstrated the positive impact of tactile feedback systems on gait stability and symmetry in lower limb amputees. Vibrotactile feedback systems can help mitigate gait asymmetry by providing real-time cues about foot–ground contact. Similarly, Martini et al. [25] showed that using vibrotactile feedback on the waist to transfer insole pressure data, participants experienced improved temporal gait symmetry, which contributed to more balanced walking patterns over time. High accuracy in detecting movement direction and force has also been shown using a pneumatic mechanotactile feedback system that utilized piezoresistive pressure sensors to capture ground contact and deliver corresponding tactile signals via balloon actuators [42]. An alternative approach for haptic feedback is a non-invasive wearable neuroprosthesis system [15], which provides real-time tactile and proprioceptive feedback to transfemoral amputees (TFAs) via electrotactile stimulation. Using an insole with a force sensor and surface electrodes on the residual limb, the system successfully conveyed knee angle and foot pressure information, resulting in increased gait symmetry and increased user confidence during daily walking tasks.

### 3.2. Balance and Postural Stability

In addition to gait improvement, haptic feedback systems can improve postural stability by delivering sensory information about the position of the prosthetic limb. Findings by Husman et al. [43] demonstrated that a wearable skin-stretch mechanotactile device might improve balance control and reduce fall risk by providing users with discrete proprioceptive feedback. Similarly, Rusaw et al. [44] examined vibrotactile feedback on transtibial amputees (TTAs), showing that vibratory cues based on center of pressure movement improved balance and postural stability. Chen et al. [45] took a similar approach, developing a sensory substitution system aimed at improving postural stability in lower-limb amputees by substituting missing foot pressure feedback with vibrotactile signals. Using pressure sensors in the insole to measure foot–ground interaction, the system delivered real-time feedback via two vibrators placed on the forearm. Participants with transtibial amputations showed reduced body sway, particularly under challenging visual disturbances, indicating that vibrotactile feedback can improve postural control and help close the sensorimotor loop for individuals with sensory impairments in the lower limbs. Emerging wearable technologies that enhance kinesthetic perception, the internal sense of body movement and position, have also shown potential in maintaining balance and postural stability [46].

### 3.3. Spatial Awareness and Proprioception

To improve foot placement perception in prosthesis users, Rokhmanova et al. [47] focused on vibrotactile feedback. Their findings indicate that vibrotactile cues significantly increase spatial awareness on uneven surfaces, which is critical for safe navigation on stairs and variable terrains. This improvement in foot placement accuracy supports more confident and stable gait patterns. Similarly, Sie et al. [48] developed a wearable haptic feedback system to assist lower-limb prosthesis users during stair descent, a challenging task due to the lack of plantar sensation. The system consists of a force-sensitive insole paired with thigh-mounted vibrotactile actuators that convey stair edge location information. The testing showed that this feedback reduced errors in detecting stair positions, potentially improving stability and user confidence on stairs. Maldonado et al. [24] developed a vibrotactile device to improve proprioception in lower-limb amputees by enhancing their ability to respond to environmental perturbations. In pilot testing, the device significantly improved participants’ reaction times to the simulated perturbations in standing and accuracy of movement during walking, demonstrating its potential as a tool for improving balance and reducing fall risk among prosthesis users. In efforts to advance control in robotic prosthetics, Chen et al. [26] combined vibrotactile feedback with volitional myoelectric control in a robotic transtibial prosthesis to provide amputees with real-time ankle position feedback. The results showed a 50% reduction in virtual ankle control error when vibrotactile feedback was included, demonstrating the effectiveness of combined sensory-motor feedback for boosting proprioception and control in robotic prosthetic limbs.

Taking a different approach, several studies have addressed bionic limbs that provide sensory feedback with electrodes implanted in peripheral nerves to provide sensory information directly to the nervous system [28,30,49,50,51,52], aiming for wider clinical use by addressing mechanical and sensory compatibility in wearable systems. These systems are aimed at creating a more natural, intuitive connection between the prosthesis and user by delivering real-time feedback. Such feedback can increase externalization and the sense of embodiment, allowing users to interact more naturally with their environment. However, challenges such as surgical risks [53], high costs, and the complexity of achieving precise signal clarity present challenges for this approach (Table 2).

### 3.4. Phantom Limb Pain

Potential effects of feedback systems on phantom limb pain via invasive and non-invasive approaches have been studied. Dietrich et al. [40] found that a leg prosthesis with feedback significantly reduced phantom limb pain in lower-limb amputees. Electrical stimulation provided foot contact sensations, which helped alleviate phantom limb pain intensity and frequency. This approach shows promise as a non-invasive method for long-term phantom pain relief in amputees, and similar results may occur for vibrotactile feedback systems. Petrini et al. [28] found that sensory feedback restoration in a leg prosthesis reduced phantom limb pain for TFAs by stimulating the tibial nerve through implanted electrodes.

## 4. Issues Regarding the Design of Haptic Feedback Systems

The design of haptic feedback systems for prosthetics has rapidly evolved. Various actuator types and feedback modalities [36,54] have been tested to optimize system performance and increase user satisfaction. System design for haptic feedback typically involves configuring actuators to deliver tactile information that is both precise and easily perceptible. This section explores the methodologies and designs implemented across studies to deliver effective feedback.

### 4.1. Actuator Placement and Feedback Modality

Different areas on the body, such as the torso and back [55,56], hand [33,57], forearm [27,45,58] and thigh (in both healthy individuals and amputees) [26,42,47], have been explored in studies on haptic feedback. Lauretti et al. [18] compared forearm and lower-back placement of vibrotactile actuators in both balance and proprioception tasks, finding no significant differences in effectiveness between the two sites, suggesting that placement can be flexibly adapted to user needs without compromising performance. Yet, no comprehensive comparison of the effectiveness of different feedback modalities between different limbs is available. However, it has been shown that responses to vibrotactile stimuli will differ across body regions [59] and this variation must be considered when designing wearable vibrotactile systems. Even though a given body part may have high sensitivity, stimulation there may not be optimal. The palms and fingertips have high tactile resolution but equipment that interferes with their use for other tasks negatively affects interaction with the environment. Similarly, although the tongue has high tactile resolution and can effectively convey information, users must be able to talk while using substitution devices [17].

The choice of feedback modality (vibrotactile, electrotactile, or mechanotactile) will influence the effectiveness of any sensory substitution device (SSD) depending on the anatomical and sensory properties of the target area [60]. While vibrotactile feedback is widely used for wearable applications due to its simplicity and energy efficiency [61], electrotactile feedback provides finer sensory discrimination by stimulating sensory nerves directly or indirectly [40,49]. However, mechanotactile feedback, where physical force is applied and the skin stretched, is particularly beneficial for proprioceptive feedback and force estimation [20]. Understanding these differences is essential for optimal haptic feedback design where actuator locations of and specific needs of the user are considered.

**Figure 2:**
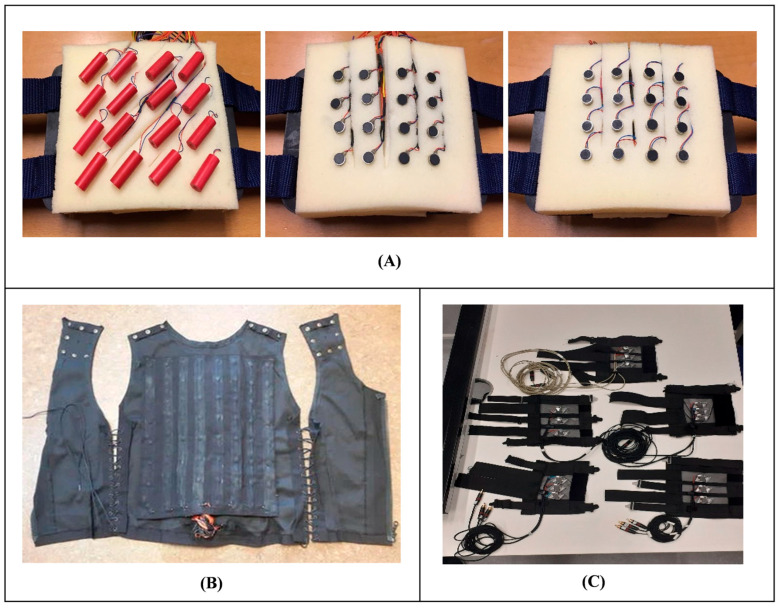
Examples of vibrotactile system configurations: (**A**) Vibrotactile stimulation array for the back with different intervals and vibrotactile actuators [62]. (**B**) Vibrotactile vest [55]. (**C**) Vibrotactile sleeves with different interspacing between actuators [58].

The design of effective vibrotactile feedback systems requires careful consideration of tactor placement, spacing, and timing. Optimal configurations may maximize the system’s ability to convey clear and accurate sensory information. But this requires a consideration of anisotropies in vibrotactile perception (Figure 2) [55,58,62]. Spatial acuity varies by the distance between tactors and different tactor types and sizes, with minimum spacing ranging from 10 mm to 20 mm, depending on the location on the body. Moreover, vibrotactile spatial acuity is higher for horizontal than for vertical stimuli, particularly on the back and torso [62]. While these spatial considerations primarily apply to vibrotactile systems, different feedback methods also present unique trade-offs. Vibrotactile feedback provides clear but generalized tactile cues, whereas electrotactile stimulation enables more localized and precise sensory feedback but requires careful calibration to avoid discomfort [29]. Meanwhile, although mechanotactile feedback is effective for force perception, it requires bulkier actuators that can limit usability in compact, lightweight prosthetics [20]. These factors must be carefully balanced based on the intended application and user needs.

Yeganeh et al. [63] highlighted that vibrotactile accuracy is often highest near joints, such as the wrist and elbow, which may act as tactile anchor points [64]. This increased accuracy near joints suggests that tactor placement in these areas could increase the amount of information conveyed, which is particularly beneficial in designing wearable devices for complex motion tasks. Additionally, Shi et al. [65] found that increased distance between tactors and increased vibration intensity improved feedback accuracy, especially for users distinguishing between patterns. Also, placing the tactors on the inner socket layer, between the socket and the user’s skin, produced the highest vibration amplitudes and improved accuracy due to direct contact with the skin. Valette et al. [29] used a multichannel electrotactile feedback in the stump of TFAs to examine walking performance. They found that dynamic walking scenarios can adversely affect the perception of electrical stimulation, which can inform future wearable prosthetic designs.

### 4.2. Perceptual Illusions

Various haptic illusions provide valuable insight into how the brain interprets spatiotemporal patterns in touch, often revealing perceptual biases that can influence how vibrotactile feedback is experienced. One well-known example is the cutaneous rabbit illusion, in which a sequence of rapid taps delivered to separate points on the skin creates the sensation of continuous motion across the space in between, although no stimulation occurs at those intermediate locations [66,67,68]. The funneling illusion occurs when two simultaneous vibratory stimuli are perceived as a single touch located midway between the actual contact points [69,70,71]. Temporal illusions such as the tau effect, where longer intervals between stimuli make spatial distances feel greater [72,73], and the kappa effect, where larger spatial distances lead to the perception of longer durations [74,75], show the tight coupling of spatial and temporal processing in the tactile domain. Similarly, the apparent tactile motion illusion, where sequential stimulation evokes a sense of continuous motion even when there is no actual displacement [76,77], underscores the brain’s tendency to impose coherent motion patterns onto discrete inputs. Hoffmann et al. [78] demonstrated how intensity variations between sequential vibrotactile stimuli can lead to systematic localization errors. Strong intensity stimuli followed by weaker ones were perceived as having downward movement, and vice versa. They called this the ‘intensity order illusion (IOI)’ and this illusion underscores the importance of carefully controlling intensity in haptic designs to reduce perceptual distortions. Makarov et al. [79] further investigated the IOI, showing that amplitude changes alone create this illusion, not changes in frequency. A second high amplitude stimulus following a weaker amplitude one was perceived as higher, while frequency variations did not affect the illusion. Another important result was that the IOI was stronger vertically than horizontally, again showing how stimulation direction affects the spatial perception of vibrotactile stimuli.

### 4.3. Technical Parameters

Should vibrotactile patterns involving more than one stimulation be presented sequentially or simultaneously? While simultaneous presentation might avoid issues like the illusions discussed above and require a shorter time for the presentation of a complete pattern, there is the danger of confusion if the stimulations are not temporally distinguished. Yeganeh et al. [80] demonstrated that the accuracy of sequential presentation of vibrotactile patterns is far higher than simultaneous presentation. This is important for designing vibrotactile feedback systems involving multi-tactor arrays, since simultaneous signals could lead to sensory overlap and confusion. It should also be noted that Lauretti et al. [18] found that continuous feedback offered smoother spatial resolution, while discrete feedback provided stronger, more localized sensations, which resulted in higher accuracy.

Frequency plays a crucial role in the effectiveness of vibrotactile feedback. Ævarsson et al. [81] found that the sensitivity to vibrotactile stimuli at the wrist peaks at specific frequency ranges. They tested sensitivity to vibrotactile patterns ranging from 25 to 1000 Hz, finding that accuracy was highest for 75–275 Hz. Then, Yeganeh et al. [63] found that frequency variations within the range between 100 and 250 Hz did not affect discrimination performance and localization accuracy. Optimal frequency and intensity combinations in haptic feedback systems could potentially improve accuracy and reduce localization errors [27,61].

### 4.4. Practical Considerations

For wearable tactile stimulation, designers of haptic feedback systems must consider additional challenges beyond sensory perception. For instance, durability plays a crucial role in prosthetic feedback systems. Repeated mechanical stress and environmental exposure can affect the durability of actuators and electronic components. Thus, encapsulating actuators in shock-absorbing [82] or water-resistant [83] materials can increase device lifespan and reliability. Additionally, power consumption is an important consideration, particularly for battery-powered prosthetic systems. Vibrotactile and electrotactile actuators are typically more energy-efficient than mechanotactile stimulators [42,61], which may require more power depending on the intensity and duration of feedback delivery. Strategies such as adaptive feedback modulation and energy-efficient actuator design can help optimize battery life without compromising feedback quality.

## 5. Devices and User Experience

Wearable tactile systems have gained popularity due to their flexibility and potential for real-time feedback. Such systems are often integrated into prosthetics or fabric-based devices like sleeves, belts, or vests, providing users with continuous haptic feedback during everyday activities. Yeganeh et al. [80] explored the use of voice coil actuators in vibrotactile sleeves on the forearm (Figure 2), demonstrating their effectiveness in conveying detailed spatial information while maintaining user comfort. Plauche et al. [84] and Wan et al. [57] further explored wearable vibrotactile feedback devices, acquiring data using insole force sensors seamlessly integrated into clothing. Their work highlighted the adaptability of vibrotactile devices for real-world scenarios, providing users with haptic cues for posture correction, balance, and movement awareness.

### 5.1. Training and User Adaptation

Training protocols can significantly increase user confidence and accelerate proficiency in interpreting haptic cues. Marayong et al. [85] discussed the role of vibrotactile feedback in rehabilitative training for individuals with lower limb amputations. Their work demonstrated that the regular use of vibrotactile devices in controlled training sessions can accelerate proprioceptive adaptation, helping users to integrate feedback more effectively into their motor control. Canino et al. [86] and Leal et al. [87] similarly examined the usability of haptic systems and found that user experience improved with feedback training. The effectiveness of tactile feedback systems is closely linked to the learning curve associated with their use [27,41]. User engagement also affects the learning curve, with frequent usage and exposure to real-world applications contributing to better skill acquisition and retention.

Users must adapt to the interpretation of haptic cues and refine their responses over time. This adaptation period varies based on factors such as individual sensory sensitivity, prior experience with feedback modality, and the complexity of the feedback system. New users of tactile feedback systems often undergo an initial adaptation phase where they familiarize themselves with different types of haptic signals and their corresponding meanings [21,25]. During this period, users may struggle with distinguishing between various tactile cues, leading to a reliance on visual confirmation or compensatory strategies. Yeganeh et al. [80] found that users’ ability to recognize vibrotactile patterns improved significantly with practice, suggesting that well-designed training protocols could increase the usability of these systems [88,89]. Early-stage users may require frequent and strong feedback to effectively interpret information. Over time, as proficiency increases, the need for intense signals may decrease, and feedback will become more seamlessly integrated into movement routines [15]. This gradual shift highlights the importance of designing systems with adjustable stimulation and other customization options to accommodate different learning paces.

### 5.2. Usability

User feedback on haptic systems can evolve significantly over multiple prototypes and extended use. Initially, users may report discomfort, cognitive overload, or difficulty in distinguishing between signals. However, as familiarity grows, preferences become more refined, leading to adjustments in signal intensity, frequency, and actuator placement. Utilizing standardized measures such as the System Usability Scale (SUS) [41,90] provides valuable quantitative data on user satisfaction and system usability, facilitating direct comparison across iterative prototypes. The regular collection of SUS scores can highlight usability improvements and pinpoint specific areas needing refinement. Notably, the shift from conscious to subconscious processing and device control marks a critical milestone in adaptation which underscores the need for iterative design approaches where both qualitative user feedback and quantitative metrics like SUS are continuously used to refine system functionality. In this context, qualitative research methods such as interviews and contextual inquiry have proven especially valuable [22,91]. These methods can uncover nuanced insights about user needs, trust in technology, perceived limitations, and psychological adaptation, factors often missed by quantitative tools alone. Building on this, Vimal et al. [92] observed that haptic feedback can increase the accuracy of the perceived position of the prosthesis and artificial limb acceptability, contributing to a more intuitive and functional experience. In Crea et al. [41], participants reported positive usability experiences from vibrotactile feedback, noting that the low-intensity, time-discrete vibrations were readily perceptible without causing discomfort, even during extended use. The systems were perceived as user-friendly and minimally intrusive, and participants were able to maintain improved gait symmetry without increased cognitive load. While these findings indicate that well-designed haptic feedback can be comfortable and intuitive, potential drawbacks must still be considered. Discomfort can arise from stimulus intensity, actuator placement, or prolonged exposure. For example, high-intensity electrotactile feedback may cause skin irritation, whereas mechanotactile feedback could lead to pressure discomfort if actuators exert excessive force. Additionally, vibrotactile feedback may lead to sensory adaptation over time, reducing its effectiveness [93,94].

### 5.3. Cognitive Load

Another important challenge is potential cognitive overload, where excessive or complex feedback can overwhelm the user’s ability to process sensory information efficiently. This is particularly relevant in multimodal feedback systems that combine multiple types of stimuli (e.g., vibrotactile + mechanotactile feedback [61] or combined tactile and auditory feedback [88,89]). If the feedback is too frequent or lacks clear interpretation cues, users may experience delayed reaction times, increased mental effort, or confusion. Note, however, that Huang et al. [61] demonstrated that combining vibrotactile and mechanotactile feedback improved recognition rates and reduced mental load for some users. Several approaches have been used to measure cognitive load in haptic feedback studies. One widely used subjective method is the NASA Task Load Index (NASA-TLX) [41,95], which is a self-reported workload assessment tool measuring mental demand, effort, frustration, and perceived performance, or the Visual Analog Scale (VAS) [30,33], which captures perceived fatigue, discomfort, or mental effort. Additionally, reaction time analysis and task accuracy measurements help determine whether users can effectively integrate haptic feedback without excessive cognitive strain. In some studies, physiological indicators such as electroencephalography (EEG)-based cognitive load monitoring [28,49,96] or functional Near-Infrared Spectroscopy (fNIRS) [32,97] are also used to assess real-time cognitive effort during haptic interactions. To mitigate discomfort and cognitive overload, adaptive feedback strategies are being explored where feedback intensity, duration, or frequency is dynamically adjusted by user responses. Such mechanisms can ensure that haptic cues remain informative without becoming intrusive or causing fatigue.

## 6. Discussion and Future Directions

The reviewed studies strongly suggest that haptic feedback systems offer a promising solution for enhancing the sensory experiences and functional performance of prosthetic limb users. Despite clear benefits, the field of haptic feedback in prosthetics faces several challenges. A critical one is standardization; the lack of uniform guidelines for actuator configurations, feedback intensity, and sensory modalities has resulted in inconsistent device performance and variable outcomes. Additionally, user-specific calibration and training requirements present barriers to large-scale clinical implementation. Moreover, there is currently no consensus on intensity measurements and calibration methods. Another challenge is the personalization of devices, adapting them to individual needs, including optimal tactor form, intensity, frequency and placement based on user preferences and physiological differences. Personalized threshold measurements may be crucial for the optimal user experiences [81]. Another overlooked challenge is the lack of comparative studies between new prosthetic users and experienced users in terms of their adaptation to haptic feedback systems. Understanding how prior experience with prosthetics influences the learning curve, interpretation of feedback, and overall system usability could inform tailored training protocols and design strategies. New users may benefit from more guided or simplified feedback initially, whereas experienced users might adapt faster or benefit from different forms of sensory input. Such comparative insights may help create adaptive systems that cater to varying levels of familiarity and functional expectations.

Yet another challenge arises in the form of durability. While current studies have demonstrated short-term benefits of feedback systems, there is a lack of long-term data on their clinical efficacy and usability [42]. Importantly, many studies are limited by small sample sizes and are typically conducted in controlled laboratory environments. Future research should focus on engaging users with haptic feedback systems over weeks or months in longitudinal studies that assess the accuracy, reaction times, durability, user adoption, and overall effectiveness of these systems in real-world settings. Addressing these challenges will be crucial for bringing feedback systems to widespread clinical use. Another challenge for haptic feedback systems is the integration of the modalities of various tactile systems with one another or with other sensory modalities, such as auditory or visual cues. By addressing this issue, multi-modal systems could provide a more comprehensive sensory experience for users, potentially improving overall prosthesis control and reducing cognitive load [61,98], such as in the Sound of Vision SSD [88,89]. Incorporating kinesthetic perception into such multi-modal approaches may further enhance human–computer interaction, rehabilitation, virtual reality, and embodied robotics [46].

The determination of optimal body locations for tactor placement is important and requires further study, as different locations might affect user comfort, perceptual clarity, and overall feedback effectiveness. Currently, there has been no comprehensive comparison of tactor placements, for example on the forearm, intact limb, or residual limb, in terms of ease of interpretation, user comfort, and practical application. Each potential tactor location has unique advantages and drawbacks for amputees. For instance, placing tactors on the forearm provides plenty of space for stimulation but may make it difficult for users to intuitively relate forearm feedback to information about the position of a prosthetic leg. Such an arrangement may feel “gadget-like” rather than essential, potentially reducing the likelihood of daily use. However, in cases of electromyography (EMG)-controlled prosthetic legs, the forearm may be a suitable option, as haptic feedback stimulations on the socket might interfere with EMG signals [99,100,101]. In contrast, placing tactors on the residual limb or stump offers a more natural sense of applying feedback directly to the prosthetic leg, enhancing the user’s sense of embodiment and the feeling that the prosthetic is part of their body [102,103,104], particularly considering promising results from prior studies [24,29,61,92] using tactors in the socket. This approach, however, is not without limitations; the design may need to account for variations in stump length [105], and users with diabetes may experience reduced sensation in this area [106,107]. Additionally, the socket pressure must be considered [65].

Diverse information about prosthetic systems may be used as input for haptic feedback systems, including EMG signals from residual muscles, joint angles, joint stiffness, and pressure or force measurements (Table 3, Table 4 and Table 5). To best utilize these inputs, the haptic feedback system’s design should emphasize a tactile arrangement that maximizes the quantity of haptic actuators while ensuring their reliable perception by users. A practical strategy could involve starting with a simple configuration and gradually increasing the complexity and quantity of actuators in later prototypes. This iterative development process may facilitate system refinement.

Considering the varied requirements of prosthetic users and the significance of customization, an adaptable feedback system that can effectively transmit numerous types of data would provide considerable benefits. This adaptability guarantees that the system can be tailored to meet specific user preferences and functional needs. Nonetheless, caution should be exercised to prevent unnecessary design complexity. Excessively complex feedback can negatively affect usability and understanding, potentially reducing the system’s advantages. Achieving a balance between delivering comprehensive, informative feedback and ensuring user-friendliness is essential for the success of these systems.

## 7. Conclusions

Haptic feedback systems in prosthetics have the potential to substantially improve the quality of life of amputees or those with visual or auditory impairments. These devices can, if designed properly, where basic research is taken into account, result in both effective and empowering devices. However, despite notable progress in this field, several challenges remain unaddressed. The lack of standardization in actuator configurations, feedback characteristics, and sensory modalities leads to variability in user experiences, limiting the widespread adoption of these technologies. Additionally, long-term clinical efficacy and usability data are scarce, making it difficult to determine the sustainability of these systems in real-world applications. Future research should focus on addressing these challenges through interdisciplinary collaboration, user-centered design, and technological advancements in feedback modalities. By developing adaptable, intuitive, and standardized haptic systems, researchers and engineers can bridge the gap between prosthetic devices and natural sensation, ultimately increasing mobility, confidence, and quality of life for amputees. The next generation of haptic feedback systems must prioritize not only functionality but also accessibility, long-term usability, and safety, ensuring that these innovations reach a broader population in both clinical and everyday settings.

## Figures and Tables

**Table 1:** Comparison of haptic feedback methods.

Haptic Feedback Method	Principle of Operation	Advantages	Challenges
Vibrotactile	Uses actuators to generate vibrations on the skin, simulating touch sensations	Compact, energy-efficient, widely studied	Limited spatial resolution, possible desensitization over time
Mechanotactile	Applies direct mechanical pressure or skin stretch to convey sensory information	Can mimic natural touch more closely, useful for proprioception	Can be bulky, actuator complexity, potential user discomfort
Electrotactile	Delivers electrical pulses to stimulate sensory nerves, creating artificial touch sensations	Can provide precise and varied sensory signals	May cause discomfort or irritation, requires careful calibration

**Table 2:** Comparison of invasive and non-invasive haptic feedback approaches for prosthetic applications.

	Invasive Approaches (Intraneural, Implanted Electrodes)	Non-Invasive Approaches (Vibrotactile, Mechanotactile, Electrotactile)
**Precision of feedback**	High (direct neural stimulation, naturalistic sensation)	Moderate to low (surface-level cues, limited spatial resolution)
**Embodiment and** **externalization**	Strong, prosthesis often perceived as part of body	Moderate, may feel “gadget-like” without extensive training
**Surgical risks**	Present (implantation required, risk of infection/complications)	None
**Long-term** **stability**	Potential issues (signal degradation, electrode encapsulation)	High (no implants, easier maintenance)
**Cost**	High (surgery, device development, maintenance)	Lower (wearable actuators, simpler electronics)
**Accessibility**	Limited to specialized clinics and research settings	Broad (portable, wearable, can be mass-produced)
**Clinical** **adoption**	Early-stage, limited trials	Already implemented in several experimental and prototype devices

**Table 3:** Overview of haptic systems: key details and study parameters by application in lower limb prosthesis.

Study	Participants	Key Findings	Limitations	Body Locations	System Source
**Electrotactile**					
Valette et al. (2023) [29]	11 AB, 3 TTA, 3 TFA	Reduced electrotactile perception, higher sensation and discomfort thresholds, lower spatial discrimination accuracy during walking	Small amputee sample size, perception variability across individuals	Thigh, residual limb	Force sensors
Basla et al. (2022) [15]	3 AB, 3 TFA	Enhanced walking symmetry	Small sample size, requires long-term and home-based assessment	Thigh, stump, hip	Knee angles, Force sensors
Dietrich et al. (2018) [40]	14 TTA	Reduced phantom limb pain, increased prosthesis functionality, and improved walking stability	Small sample size, short training duration, feedback system design limitations	Thigh	Force sensors
**Intraneural electrodes**					
Preatoni et al. (2021) [30]	1 TFA	Sensory feedback reduced perceived prosthesis weight, increased embodiment and confidence, and maintained walking speed under cognitive load	Single-subject study	Residual limb	Force sensors
Petrini et al. (2019) [28]	2 TFA	Improved walking speed, reduced metabolic cost, increased confidence, and decreased phantom limb pain	Small sample size, limited duration of the study (3 months), need for larger trials to assess long-term clinical benefits	Residual limb	Knee angles, force sensors
Petrini et al. (2019) [49]	3 TFA	Improved mobility, fall prevention, agility, embodiment, and reduced cognitive load	Small sample size, short study duration (3 months per subject), non-implantable system tested in a lab setting	Residual limb	Knee angles, force sensors
**Mechanotactile**					
Husman et al. (2016) [43]	3 AB	High perceptibility for balance control	Tested only in static conditions, no amputee participants	Thigh	Inertial measurement unit (IMU)
Canino et al. (2016) [86]	2 AB	Sustained pressure feedback significantly improved EMG control in absence of visual feedback, vibration feedback less effective due to desensitization	Vibrotactile feedback prone to desensitization, more complex trajectories require improved magnitude encoding	Thigh	EMG
Fan et al. (2008) [42]	6 AB	Demonstrating its potential to improve balance and gait in prosthetic users	Not tested on amputees, requires further optimization for portability and clinical use	Thigh	Force sensors
**Vibrotactile**					
Leal et al. (2022) [87]	2 TFA	Participants achieved high accuracy in interpreting directional and intensity-based haptic feedback	Only 2 participants, limited generalizability, COVID-19 disrupted further testing	Arm, thigh	Force sensors
Martini et al. (2021) [25]	3 TFA	Improved temporal gait symmetry with bilateral feedback, one subject retained improvement post-training	Small sample size, variability in individual responses	Waist	Force sensors
L. J. Chen et al. (2021) [45]	8 AB, 7 TTA	Improved postural stability, reducing body sway	Tested only in a controlled lab, limited to TTA, used only center of pressure as stability measure	Forearm	Force sensors
Vimal et al. (2020) [92]	5 TFA	Improved limit of stability, particularly with movable ankle joints	Only anterior–posterior center of pressure analyzed, ankle joint condition not randomized	Residual limb	Force sensors
Rokhmanova et al. (2019) [47]	10 AB, 2 TTA	Improved foot placement awareness	Only tested vibrotactile not modality-matched (pressure) feedback	Thigh	Force sensors
Shi et al. (2019) [65]	10 AB, 3 TFA	Inner socket tactors improved perception, higher intensity and spacing enhanced accuracy	Tested under static conditions (sitting), needs real-world mobility assessment	Thigh, residual limb	Simulator
Sie et al. (2018) [48]	28 AB	Improved step-edge detection, reduced localization error in visually obstructed stair descent tasks	Tested only on able-bodied subjects, possible sensor bias from boot placement and sole curvature, actuator calibration inconsistencies	Thigh	Force sensors
Lauretti et al. (2017) [18]	16 AB, 1 (TFA, TTA)	Capable of improving postural control and knee-joint proprioception	Needs further validation on a larger amputee population	Forearm, low back	Knee angles, force sensors
Maldonado et al. (2017) [24]	2 TTA	Improved proprioception and postural control; 17% faster response time in trained amputee	Small sample size, device design limitations	Thigh	Knee angles
Crea et al. (2017) [41]	3 TFA	Improved temporal gait symmetry after training, even under dual-task conditions	Small sample size, short follow-up, limited generalizability	Lower abdomen	Force sensors
B. Chen et al. (2016) [26]	8 AB, 2 TTA	Improved the perception of ankle joint position and enhances prosthetic control	Tested only in seated conditions	Thigh	Ankle angles
Wan et al. (2016) [57]	8 AB, 2 TFA, 3 TTA	Improved amputees’ ability to identify different floor conditions	Limited to standing conditions only	Hand	Force sensors
Plauché et al. (2016) [84]	9 AB	Reduced stride length, step width, and trunk sway variability, indicating improved gait stability	Tested only on able-bodied users with simulated prosthesis	Thigh	Force sensors
Canino et al. (2016) [86]	2 AB	Sustained pressure feedback significantly improved EMG control in absence of visual feedback, vibration feedback less effective due to desensitization	Vibrotactile feedback prone to desensitization, more complex trajectories require improved magnitude encoding	Thigh	EMG
Marayong et al. (2014) [85]	1 TTA	Participant could perceive and distinguish feedback types	Delay between activation and actuator output, inaccurate knee angle readings affected timing	Residual limb	Knee angles
Rusaw et al. (2012) [44]	24 TTA	Improved postural stability	Feedback only responded to normal (vertical) forces, not shear forces; feedback only provided on the prosthetic side	Thigh	Force sensors

AB: Able-bodied, TFA: transfemoral amputee, TTA: transtibial amputee.

**Table 4:** Overview of haptic systems: key details and study parameters by application in upper limb prosthesis.

Study	Participants	Key Findings	Limitations	Body Locations	System Source
**Electrotactile**					
Chai et al. (2022) [21]	10 AB, 2 TRA	Improved grip force control and stiffness recognition	No intraneural feedback due to lack of amputees, stiffness discrimination should include object deformation data	Forearm	Force sensors
**Intraneural electrodes**					
George et al. (2019) [51]	1 TRA	Improved object manipulation, grasp control, and prosthesis usability	Limited patient time, daily living activities not tested with biomimetic feedback	Residual limb	Force sensors
D’Anna et al. (2019) [52]	2 TRA	Improved task performance in prosthetic hand use, participants were able to identify object size	Limited to two channels of proprioceptive feedback, does not cover all five fingers, future research needed for wrist and elbow feedback	Residual limb	Fingers angles, Force sensors
Valle et al. (2018) [50]	2 TRA	Improved sensation naturalness, tactile sensitivity, manual dexterity, and prosthesis embodiment	Case study with a small sample size, findings need validation in a larger population	Residual limb	Force sensors
**Mechanotactile**					
Shehata et al. (2020) [33]	21 AB	Improved embodiment (ownership and location) with synchronous feedback, asynchronous feedback reduced agency	Tested only on able-bodied participants, subjective embodiment measures	Hand	Force sensors
Rossi et al. (2019) [20]	43 AB, 1 TRA	Provided accurate proprioceptive feedback on hand aperture	Large device size, not integrated into prosthetic socket	Forearm	EMG
Huang et al. (2017) [61]	3 TRA	Improved localization and intensity recognition	System requires miniaturization, high power consumption	Residual limb	Force sensors
**Vibrotactile**					
Marinelli et al. (2024) [19]	10 AB (control group), 10 AB, 4 TRA	Compact feedback with fewer motors, amputees performed similarly to able-bodied participants	Test and control conditions in separate groups of participants, long single session affects mental fatigue	Forearm	Wrist angles, EMG
Thomas et al. (2021) [32]	10 AB	Haptic feedback improved stiffness discrimination accuracy and reduced cognitive load (measured via fNIRS)	Tested only on able-bodied participant, task was simple and may not generalize to real-world use	Upper arm	Force sensors
Fontana et al. (2018) [16]	30 AB	94% accuracy in finger sensation discrimination, 85% in grasping pattern recognition	Tested only on able-bodied subjects, needs validation with amputees	Arm	Simulator
Huang et al. (2017) [61]	3 TRA	Improved localization and intensity recognition	System requires miniaturization, high power consumption	Residual limb	Force sensors
Erwin et al. (2015) [27]	8 AB	Improved virtual wrist positioning accuracy compared to no feedback	Not tested on amputees, feedback limited to one degree of freedom	Forearm	EMG

AB: Able-bodied, TRA: transradial amputee.

**Table 5:** Overview of haptic systems: key details and study parameters by application in sensory substitution design.

Study	Participants	Key Findings	Limitations	Body Locations	System Source
**Vibrotactile**					
Plaisier et al. (2024) [56]	13 AB	Spatial acuity on the back is significantly higher in the horizontal direction than in the vertical direction	N/A	Back	Simulator
Yeganeh et al. (2024) [80]	8 AB	Sequential stimulation had higher accuracy than simultaneous, with better performance for shorter patterns and learning effects over time.	Differences in timing between conditions need further study	Forearm	Simulator
Amann et al. (2024) [98]	31 AB	Participants learned to interpret vibrotactile cues and integrated them with visual info to improve accuracy	Performance varied across participants, limited training, potential skin vibration overlap	Arm	Simulator
Yeganeh et al. (2023) [58]	8 AB	20 mm was identified as the optimal interspacing for voice coil actuators on the forearm	N/A	Forearm	Simulator
Yeganeh et al. (2023) [63]	8 AB	Placing actuators near the wrist and elbow improves accuracy, frequency variations have minimal effects	Need to determine the impact of anisotropies in vibrotactile localization and the effect of denser actuator placements near anatomical landmarks	Forearm	Simulator
Makarov et al. (2023) [79]	17 AB	The intensity order illusion is caused by amplitude changes rather than frequency differences; the illusion occurs in the vertical direction but not in the horizontal direction	N/A	Waist	Simulator
Ævarsson et al. (2022) [81]	30 AB	Sensitivity was in higher frequency on the inner wrist, suggest need for personalized calibration	Further testing is needed due to demographic imbalances (e.g., age/ gender distribution)	Wrist	Simulator
Hoffmann et al. (2019) [78]	16 AB	Varying the temporal and intensity order of vibrotactile stimuli causes systematic localization errors; strong-to-weak stimuli increase downward perception and vice versa	Frequency and amplitude are linked, further research is needed to test other body parts and determine optimal parameters	Low back	Simulator
Hoffmann et al. (2018) [62]	17 AB	Spatial acuity depends on tactor type, better discrimination accuracy for horizontal presentation and normal eccentric rotating mass tactors	Limited to lower thoracic region, different tactor types under load may yield varying results	Low back	Simulator
Johannesson et al. (2017) [55]	30 AB	Spatial acuity for vibrotactile stimuli on the torso is below 13 mm; accuracy decreased as inter-tactor spacing decreased	Need to compare different tactor types	Back	Simulator

AB: Able-bodied. N/A indicates that the corresponding information was not reported in the original study.

## Data Availability

Not applicable.

## Abbreviations

The following abbreviations are used in this manuscript:

sEMG	Surface Electromyography
AB	Able-Bodied
TFA	Transfemoral Amputee
TTA	Transtibial Amputee
TRA	Transradial Amputee
SSD	Sensory Substitution Device
IOI	Intensity Order Illusion
SUS	System Usability Scale
NASA-TLX	NASA Task Load Index
VAS	Visual Analog Scale
EEG	Electroencephalography
fNIRS	functional Near-Infrared Spectroscopy
IMU	Inertial Measurement Unit
